# Supplementary material for: A study on the impact of enterprise digital transformation on informed trading
Source: PLoS One. 2024 Dec 27;19(12):e0313623. doi: 10.1371/journal.pone.0313623 (PMC11676922; doi:10.1371/journal.pone.0313623)
Supplement: S1 Appendix — (DOCX) [file pone.0313623.s001.docx]

S1 Appendix. Digital transformation indicator system

| Tier 1 | Tier 2 | Weight |
| --- | --- | --- |
| Strategic leadership | Management digital job creation | 0.083 |
|  | Management is digitally innovation-oriented and forward-looking | 0.095 |
|  | Management Digital Innovation Orientation Continuity | 0.065 |
|  | Breadth of management's digital innovation orientation | 0.045 |
|  | Strength of management's digital innovation orientation | 0.058 |
| Technology-driven | AI technology | 0.089 |
|  | Blockchain technology | 0.021 |
|  | Cloud Computing Technology | 0.03 |
|  | Big data technology | 0.022 |
| Organizational empowerment | Digital Capital Investment Program | 0.049 |
|  | Digital Workforce Input Program | 0.025 |
|  | Digital infrastructure development | 0.012 |
|  | Science and technology innovation base construction | 0.012 |
| Environmental support | Number of patents for inventions in the industry | 0.007 |
|  | R&D activities in the industry | 0.006 |
|  | New product development and sales in your industry | 0.005 |
|  | Intensity of digitization technology in your industry | 0.004 |
|  | Intensity of digital capital investment in your industry | 0.004 |
|  | Intensity of human capital investment in the industry | 0.003 |
|  | Density of fiber optic cables in the city | 0.002 |
|  | Mobile switch capacity in your city | 0.001 |
|  | Scale of Internet broadband access users in your city | 0.001 |
|  | Size of mobile Internet users in your city | 0.001 |
| Digital achievements | Digital Innovation Standards | 0.1 |
|  | Digital Innovation Essay | 0.032 |
|  | Patents for digital inventions | 0.064 |
|  | Digital Innovation Qualification | 0.04 |
|  | Digital National Awards | 0.036 |
| Digital applications | Technological innovation | 0.056 |
|  | Process Innovation | 0.021 |
|  | Business Innovation | 0.011 |
